# Supplementary material for: Decadal changes in Atlantic overturning due to the excessive 1990s Labrador Sea convection
Source: Nat Commun. 2023 Aug 2;14:4635. doi: 10.1038/s41467-023-40323-9 (PMC10397207; doi:10.1038/s41467-023-40323-9)
Supplement: Supplementary file 1 — Supplementary information [file 41467_2023_40323_MOESM1_ESM.pdf]

Supplementary Information supporting the article:

Decadal changes in Atlantic overturning due to the excessive 1990s Labrador Sea convection

C.W. Böning[1,2], P. Wagner[1], P. Handmann[1,3], F. U. Schwarzkopf[1], K. Getzlaff[1] & A. Biastoch[1,2]

[1] GEOMAR Helmholtz Centre for Ocean Research Kiel, Kiel, Germany

[2] Faculty of Mathematics and Natural Sciences, Christian Albrechts Universität zu Kiel, Kiel, Germany

[3] Present address: Lhyfe, Nantes, France

Corresponding Author: C. W. Böning (cboening@geomar.de)

DOI: 10.1038/s41467-023-40323-9

Supplementary Figure 1:

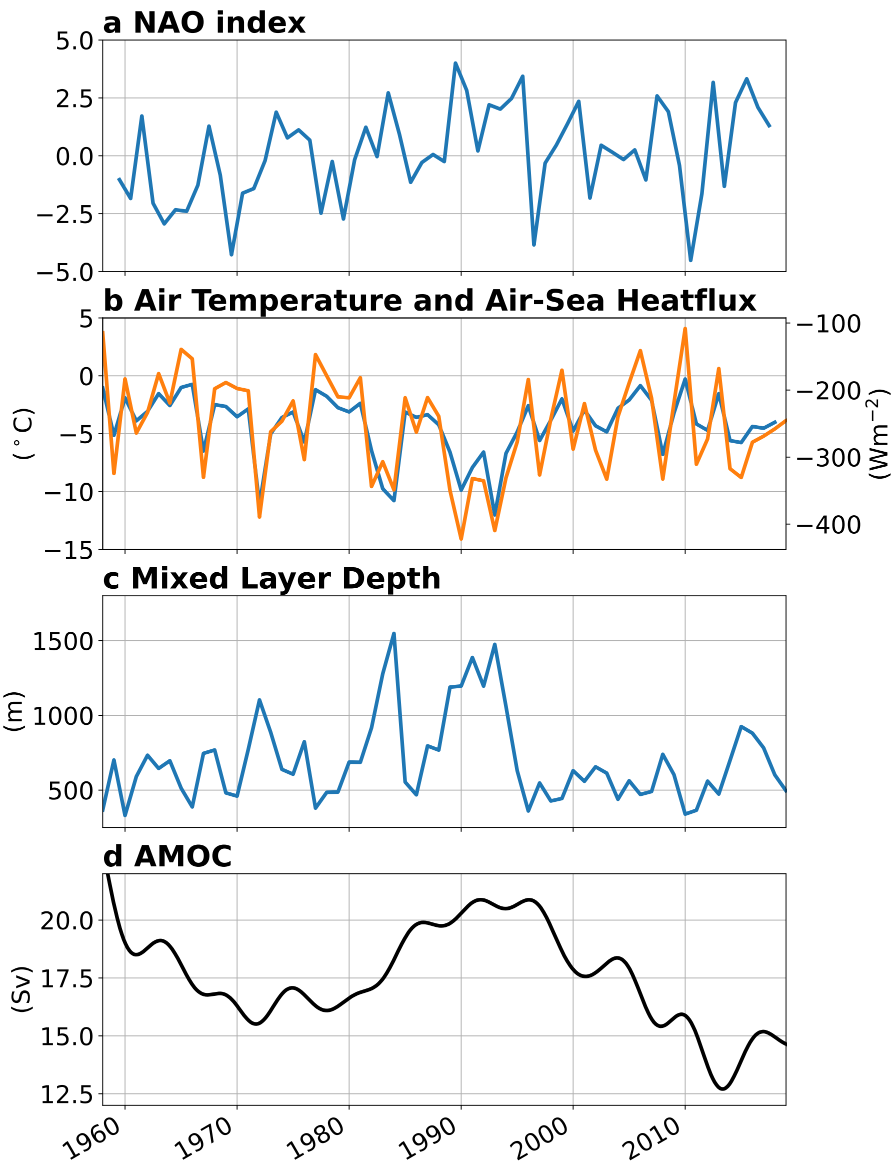

Supplementary Figure 1. Time series of properties relevant for the choice of the 1980-1981 period (shaded) for the perturbation experiment SENS: (a) North Atlantic Oscillation (NAO) index and (b) air temperature (blue curve) over the Labrador Sea box (blue frame in Fig. 1), both as given by the JRA55-do forcing data; (b), (c), (d) air-sea heat flux (orange) over the same box, winter mixed layer depth for the area as used in Fig. 2a, and AMOC transport at section 48N, all as simulated in CTRL.

Supplementary Table 1:

**Experiment**<br><br>(internal notation)	**Forcing**	**Initialisation**	**Integration period**	**Runoff**	**SSSR**<br>Piston velocity<br><br>Mask around Greenland
**CTRL**<br><br>(KFS003)	JRA55-do v1.4	WOA13	1958-2019	JRA55-do	50 m/yr<br><br>Mask
**CORE**<br><br>(KKG36013H)	CORE v2	WOA13 and 30-year spin-up	1958-2009	Climatol.	12.2 m/yr<br><br>No mask
**JRA-cr**<br><br>(KKG36101H)	JRA55-do v1.3	CORE 1979/12/31	1980-2018	Climatol.	12.2 m/yr<br><br>No mask
**JRA-short**<br><br>(KKG36107B)	JRA55-do v1.4	CORE 1979/12/31	1980-2019	JRA55-do	12.2 m/yr<br><br>Mask

Supplementary Table 1: **Overview of hindcast experiments.** Summary of CTRL and supplementary experiments, providing an overview of the main differences in the forcing product, integration period, and choices for the freshwater treatment of potential relevance to the simulation of subpolar North Atlantic variability. Climatological runoff refers to a monthly climatology, instead of the interannually varying, daily runoff data of JRA55-do. In experiments with climatological runoff, surface freshwater fluxes are provided as interannually varying monthly fields while their frequency is 3-hourly in the other experiments. Sea surface salinity restoring (SSSR) is applied with two different choices of piston velocity; in two experiments SSSR is suppressed in an 80 km wide band around Greenland (Mask), in the other two no suppression is applied (No mask). In addition to the piston velocity, CTRL differs from the other experiments by employing a correction scheme for the surface freshwater fluxes that enforces a globally balanced freshwater budget correction at each time step. In addition to the short names used in this manuscript, the internal names used for referencing these experiments in the Kiel modelling group are provided. (The internal notation for experiment SENS is JRA_SMF_KPW001.)

Supplementary Figure 2:

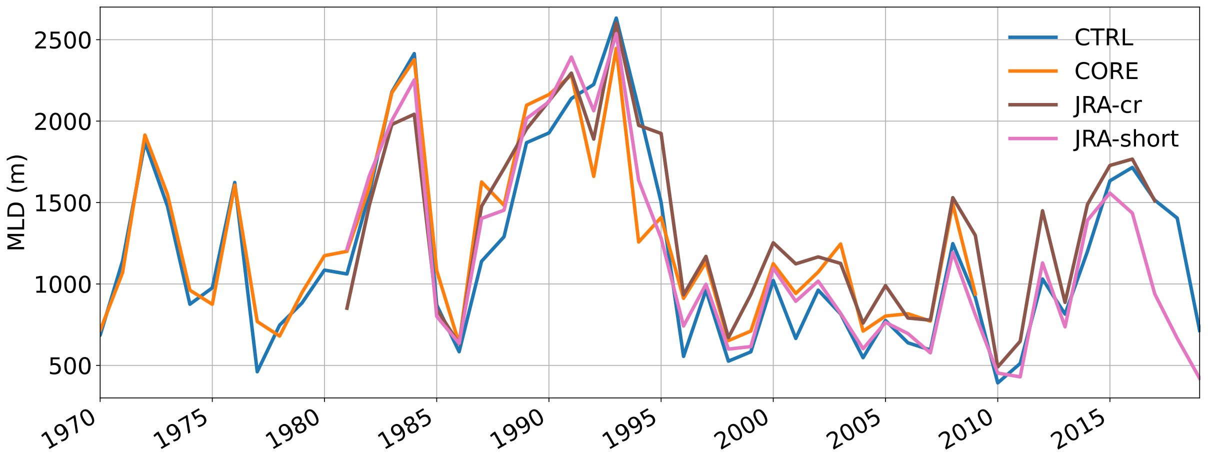

Supplementary Figure 2. Depth of the winter mixed layer in the Labrador Sea as computed in Fig. 2a for CTRL and SENS, shown here for the supplementary hindcasting experiments in comparison to CTRL.

Supplementary Figure 3:

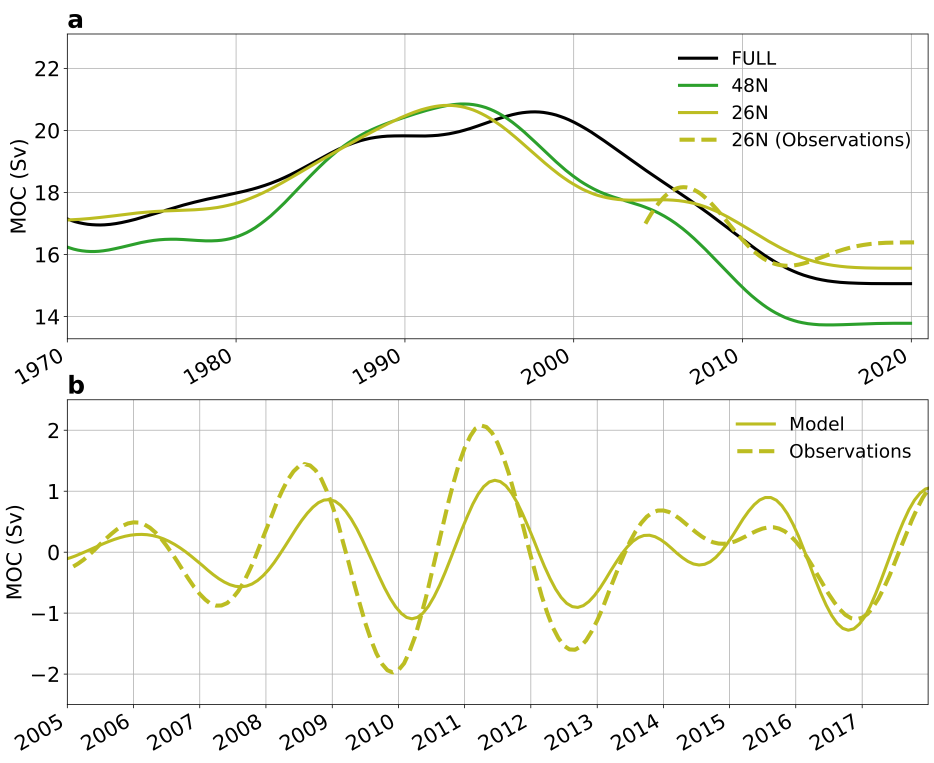

Supplementary Figure 3. AMOC time series simulated in CTRL in the subtropical North Atlantic (26N) in comparison to the subpolar sections FULL and 48N, and to the RAPID observational product. Panel (a) shows 10-yr low-pass filtered time series; the time series in panel (b) are 2-5-year band pass filtered.

Supplementary Figure 4:

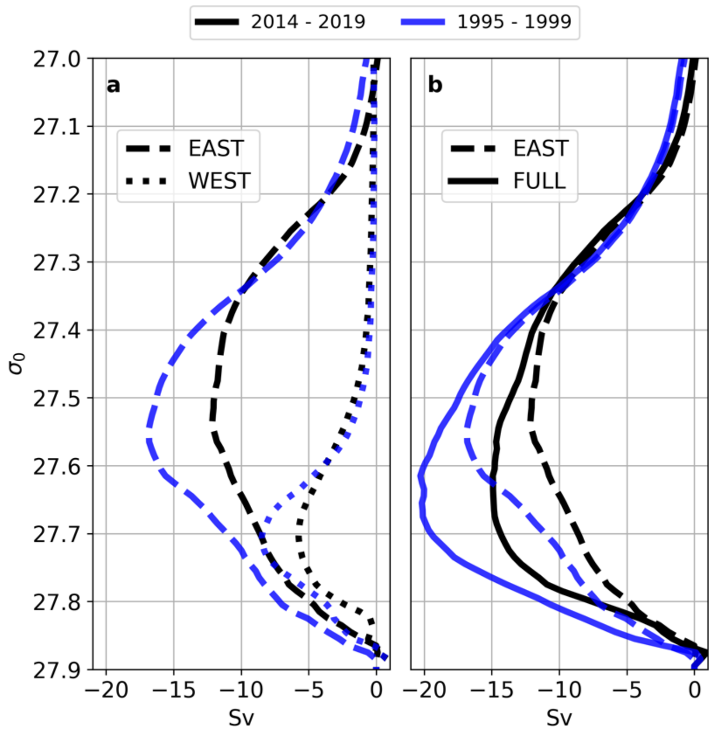

Supplementary Figure 4. Profiles of the overturning streamfunction as function of potential density (σ_0; kg m^{-3}) for the sections WEST, EAST and FULL in experiment CTRL averaged over the periods 2014-2019 (black) and 1995-1999 (blue). The location of the minima of the streamfunction profiles represent the densities which separate the northward and southward limbs of the zonally-integrated overturning circulation for these sections. **Panel a** shows that (as noted in the OSNAP observation, cf. refs.17 and 18) these densities differ between EAST and WEST, implying a compensation of the southward flows (in EAST) and northward flows (in WEST) in the range between these densities. Accordingly, the overturning transport across the western subsection, although not small individually (~6 Sv in the 2014-2019 period, and ~8 Sv during 1995-1999), merely serves to increase (see **panel b**) the density threshold between the upper and lower limb from EAST to FULL, but contributes to the FULL overturning by just 2-3 Sv during both periods.

Supplementary Figure 5:

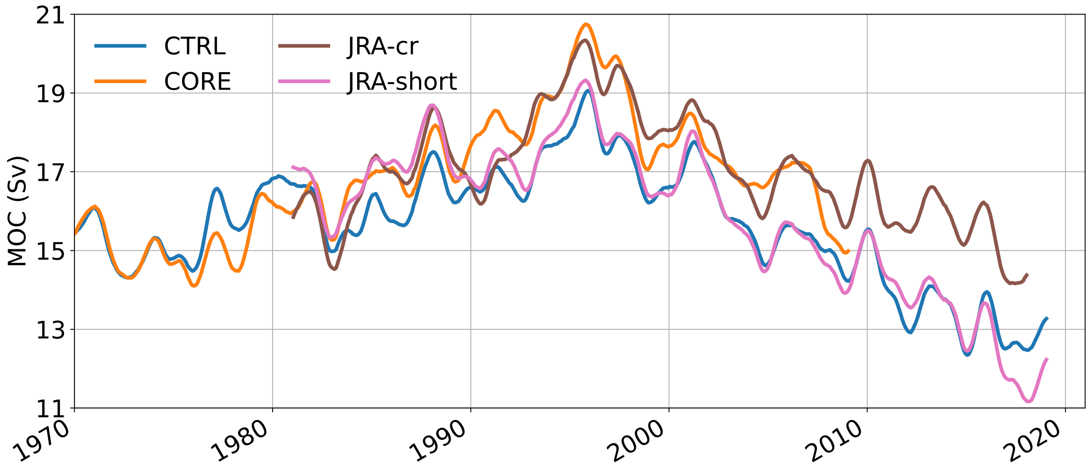

Supplementary Figure 5. Evolution of the overturning transport at EAST for the supplementary experiments in comparison to CTRL. The experiments concur in the simulation of a transport peak during 1996-1997. The increase from the 1970s to the mid-1990s is stronger in CORE compared to CTRL. The similarity of CORE and JRA-cr in this regard points to the continental runoff as a main factor for the inter-decadal evolution of the transport. The transport time series in JRA-short is similar to CTRL, indicating a negligible influence of spin-up transients except for the first ~10 years after initialisation.

Supplementary Figure 6:

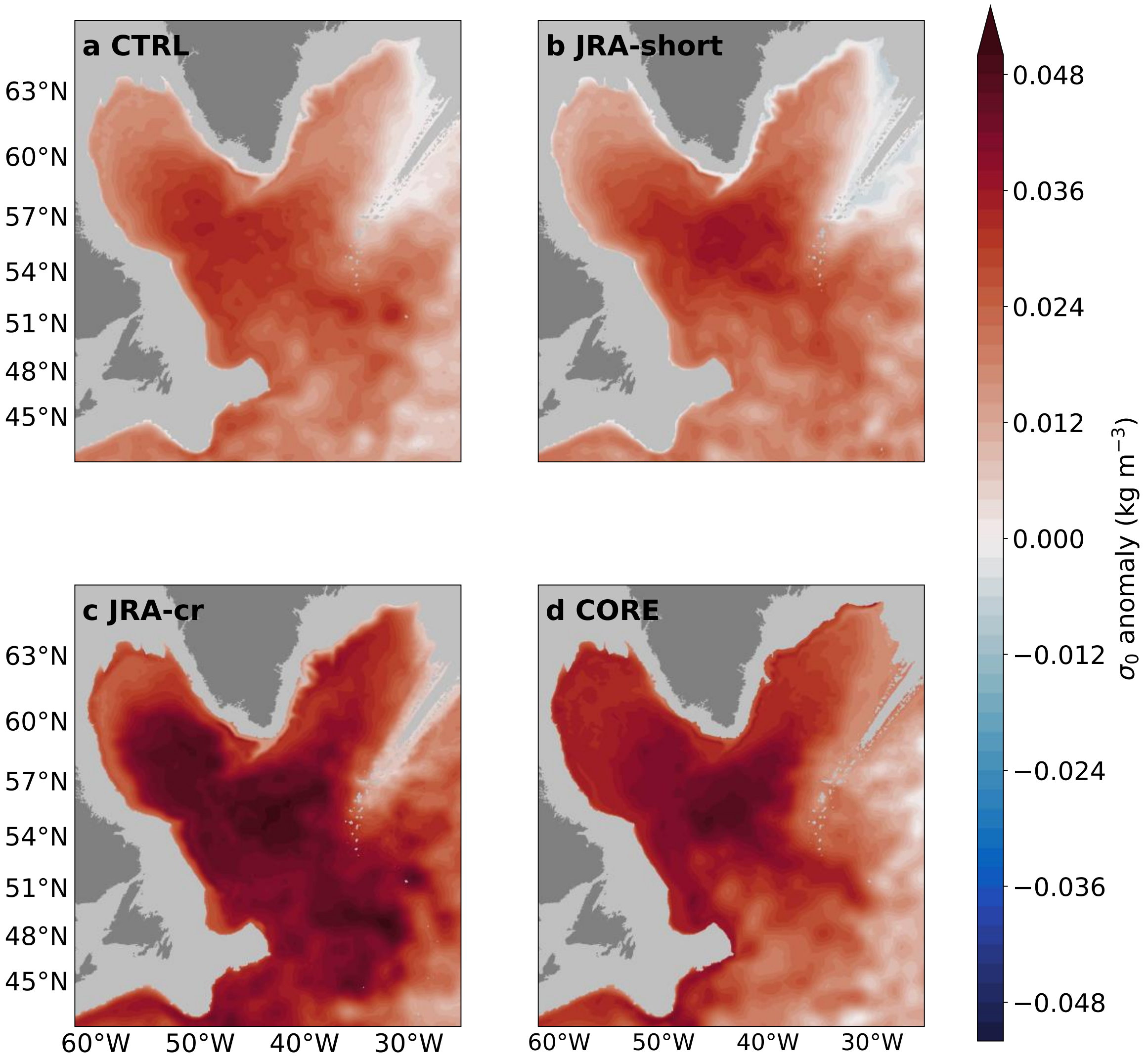

Supplementary Figure 6. Spreading of the LSW anomaly in the four hindcasting simulations, illustrated by the changes of potential density (σ_0; kg m^{-3}) at 1500 m depth between the periods 1980-1985 and 1995-2000. The maps show spreading patterns in the experiments; the amplitude of the anomaly in the Irminger Sea is somewhat larger in CORE and JRA_cr compared to CTRL and JRA_short (cf. the time series provided in Supplementary Fig. 7), indicating an influence of the differences in the specification of freshwater forcing on the evolution of the density anomaly in the LSW layer.

Supplementary Figure 7:

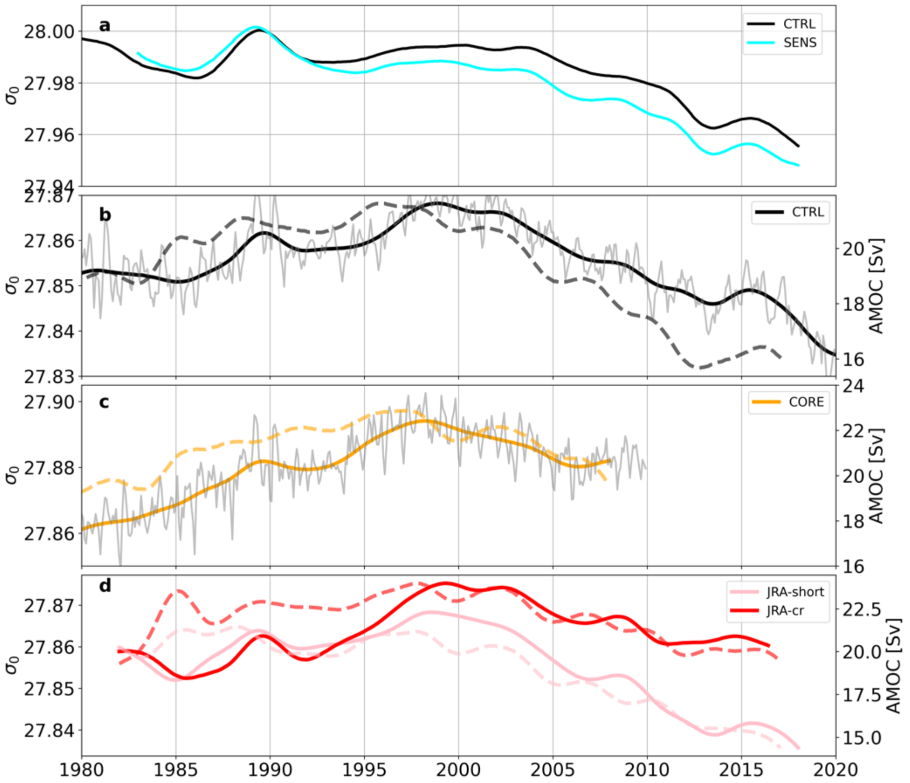

Supplementary Figure 7. (a) Temporal changes in the potential density maximum above the Denmark Strait sill in CTRL and SENS, illustrating the contrasting density evolution of the overflow and of the deep western boundary current off southeastern Greenland shown in Fig. 7. The density is relatively constant during the 1980s and 1990s in both experiments. In CTRL (in contrast to SENS, see Fig. 7) the mixing of the outflow with the increasingly dense LSW led to an increasing density trend in the deep western boundary current along southeastern Greenland between the 1970s and 1990s. After ~2000 the overflow density, and with it the density of the deep western boundary current (Fig. 7), show declining trends in both CTRL and SENS, probably related to a spurious freshening of the Nordic Seas that was noted in long-term simulations under JRA55-do forcing (ref. 19). Taken together, the density changes in the deep western boundary current may be regarded as a manifestation of two main effects: changes in the source waters at the overflow sill, and changes in the density of the ambient waters entrained into the boundary current in the Irminger Basin. While the impact of the latter is highlighted by the different overturning changes in CTRL and SENS, the former is common to both experiments and represents a likely cause of the declining AMOC trends during the last decades of the two experiments. Panels (b) to (d) supplement Fig. 7 by showing the AMOC transport at FULL (dashed lines) in relation to the density changes (1500 – 2000 m average) at position 5 (continuous lines) for all hindcasting experiments. All time series are 4-yr low-pass filtered; in (b) and (c) the filtered density time series are supplemented by depictions of the monthly varying values (grey). In contrast to the indication of a co-evolution between AMOC and wbc density in the low-pass filtered time series, there is no relation (r = 0.3) between the density and overturning variability at higher frequencies.
